# Outperforming hummingbirds’ load-lifting capability with a lightweight hummingbird-like flapping-wing mechanism

**DOI:** 10.1242/bio.014357

**Published:** 2016-07-21

**Authors:** Frederik Leys, Dominiek Reynaerts, Dirk Vandepitte

**Affiliations:** Department of Mechanical Engineering, Katholieke Universiteit Leuven, Celestijnenlaan 300, Box 2420, Leuven 3001, Belgium

**Keywords:** Rufous hummingbird, Trochilidae, Flapping, Wing, Mechanism, Stroke-cam, Lightweight

## Abstract

The stroke-cam flapping mechanism presented in this paper closely mimics the wing motion of a hovering Rufous hummingbird. It is the only lightweight hummingbird-sized flapping mechanism which generates a harmonic wing stroke with both a high flapping frequency and a large stroke amplitude. Experiments on a lightweight prototype of this stroke-cam mechanism on a 50 mm-long wing demonstrate that a harmonic stroke motion is generated with a peak-to-peak stroke amplitude of 175° at a flapping frequency of 40 Hz. It generated a mass lifting capability of 5.1 g, which is largely sufficient to lift the prototype's mass of 3.39 g and larger than the mass-lifting capability of a Rufous hummingbird. The motor mass of a hummingbird-like robot which drives the stroke-cam mechanism is considerably larger (about five times) than the muscle mass of a hummingbird with comparable load-lifting capability. This paper presents a flapping wing nano aerial vehicle which is designed to possess the same lift- and thrust-generating principles of the Rufous hummingbird. The application is indoor flight. We give an overview of the wing kinematics and some specifications which should be met to develop an artificial wing, and also describe the applications of these in the mechanism which has been developed in this work.

## Abstract (precis)

**Summary:** The stroke-cam flapping mechanism closely mimics the wing motion of a hummingbird. It is the only lightweight hummingbird-sized flapping mechanism which generates a harmonic wing stroke with both a high flapping frequency and a large stroke amplitude.

## INTRODUCTION

### Indoor NAVs: agility, size and payload

Recent innovations in microelectronics, material science and mechanical miniaturization such as small MEMS accelerometers and gyroscopes, LiPo batteries and efficient micro-mechanical motors, have made it possible to develop flight vehicles at the so-called nano-scale; nano air vehicles or NAVs with a wingspan small enough to fly indoors.

These NAVs can be used for indoor missions such rescue, surveillance, security, inspection of calamities in contaminated spaces or any other first responder applications.

NAVs designed for indoor use face certain design constraints concerning size, weight and agility. The size is restricted by the limited space available indoors. Regardless of the design concept and the driving mechanism of the NAV and weight optimisation of its components, the wingspan of any flight vehicle always sets a natural limit to the maximum airborne weight of the NAV. Finally the NAV should have sufficient agility to avoid all kinds of obstacles and fly swiftly through corridors, rooms and windows.

### Flapping wings

In general, three types of NAVs can be distinguished: fixed wing NAVs, rotary wing NAVs, like helicopters or multi-copters, and flapping wing NAVs (or FNAVs), which flap their wings in order to fly just like hummingbirds and insects do. With the particular constraints of indoor flight, fixed wing NAV are usually inappropriate as their agility is very limited and more in particular, they are unable to hover. Both rotary and flapping wing NAVs offer viable alternatives. While rotary wing NAVs are well established, the Nano Hummingbird (Keennon et al., 2012) is still the only controllable flapping wing NAV able to fly untethered, next to some clapping wing NAVs like the Delfly micro (de Croon et al., 2009; http://www.delfly.nl/micro.html).

Small hummingbirds are able to perform all kinds of remarkable flying manoeuvres. They can fly forwards, backwards, sideways, hover and they can even transition swiftly between these flight regimes. They are the living proof that flapping wing propulsion is a promising choice for agile artificial NAVs.

Several authors have investigated the mechanisms and aerodynamic phenomena with small birds and insects in different flight regimes. Dickinson (1999), Ellington (1994), Aono et al. (2008) and Warrick et al. (2009) distinguish four thrust-enhancing aerodynamic phenomena that are responsible for the exceptionally high thrust generated by small flapping wings; leading edge vortex stabilization, advanced wing rotation, wake capture and added mass effect. However, a specific and complex wing motion is required to be able to take advantage of these lift-enhancing phenomena.

The complexity required to artificially generate the wing motion of hummingbirds has limited the successful development of operational FNAVs up to now to a single development (Keennon et al., 2012).

### Rufous hummingbird

The proposed wing-flapping mechanism generates a wing motion which is based on the wing motion of the Rufous hummingbird (*Selasphorus rufus*). The Rufous hummingbird is the only small hummingbird species of which the wing motion is already thoroughly studied (Tobalske et al., 2007). In addition, the wings of this bird are small enough and they flap fast enough to take advantage of at least some of the low Reynolds number lift-enhancing aerodynamic phenomena which are described above and which larger species do not take advantage of. Despite the small size of its wings, the mass of the Rufous hummingbird is about 3.4* g*. This mass is relatively high compared with other hummingbirds, which enables the development of a bio-inspired FNAV based on a design spec for the total mass in the same order of magnitude as the Rufous hummingbird.

### Hummingbird flight at hover

#### Wing kinematics

Small hummingbirds and many insects show remarkably similar wing kinematics during hovering mode. They flap their wings back and forth in a plane which is approximately horizontal, rather than up and down, like larger birds do.

**Fig. 1.**
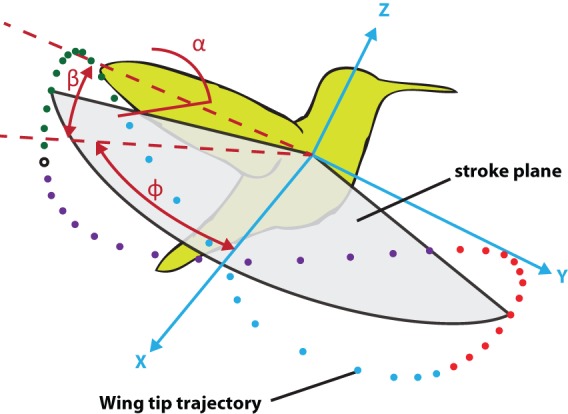
**Flapping wing kinematics.** Three angles are sufficient to fully describe the wing motion relative to the stroke plane. These angles are: the stroke angle (ϕ), the pitch angle (α) and the deviation angle (β). The coloured dots illustrate the forward stroke (blue), the pronation (red), backward stroke (purple) and supination (green). Taken and altered from Phillips and Knowles, (2011).

One flapping cycle has four consecutive phases: forward stroke, supination, backward stroke and pronation as shown in Fig. 1. During supination and pronation the wing rotates around its longitudinal axis in order to maintain a proper angle of attack at all times.

Measurements on the wing motion of the Rufous hummingbird (Tobalske et al., 2007) have revealed a complex wing motion; while hovering, the Rufous hummingbird flaps its wings at a flapping frequency of about 44* *Hz with a wingbeat amplitude of 112° in a stroke plane slightly tilted forward (about 14° from the horizontal). The angle of attack variation is described relatively to the body orientation by the chord angle and is about 92° during upward (backward) wing motion and about −38° during downward (forward) wing motion.

#### Thrust

The total force generated by the flapping wing is resolved into two components, which are defined with respect to the average stroke plane. By definition, thrust is the component of force perpendicular to the average stroke plane. A literature study reveals that three features of the wing motion have a significant influence on the thrust generated by a flapping wing: the flapping frequency (Phillips and Knowles, 2011; Shyy et al., 2010), the wingbeat amplitude (Phillips and Knowles, 2011; Shyy et al., 2010) and the angle of attack. Two other wing motion characteristics affect the magnitude of thrust, be to a much lesser extent: the speed of the wing pitch during pronation and supination (Phillips and Knowles, 2011) and the phase between stroke and wing pitch (Phillips and Knowles, 2011; Dickinson, 1999; Shyy et al., 2010).

To maximize the thrust generated by an artificial flapping wing, three parameters should be set at a high value; the flapping frequency, wingbeat amplitude and the speed of the wing rotation. At the same time, a relatively high angle of attack should be maintained during forward and backward.

The phase between the stroke (sweeping) motion of the wing and the pitching motion of the wing may also be of importance because it may have a small but significant influence on the thrust generated by a flapping wing. If a larger part of the wing pitch occurs before the stroke reversal, it is called an advanced wing pitch, while if a larger part of the wing pitch occurs after the stroke reversal it is a delayed wing pitch. Research on *Drosophila* (Dickinson, 1999) shows that a delayed wing pitch is disadvantageous for thrust generation. Also at hummingbird scale the timing of the wing pitch is of considerable importance, although at this scale other aerodynamic phenomena are in play (Warrick et al., 2009).

### Experimental research

The development of an artificial flapping wing mechanism that generates the wing motion of hummingbirds and insects is particularly challenging as a high frequency and at the same time large amplitude motion should be generated with lightweight components which are inevitable fragile. Much of the experimental research on flapping wings is restricted to dynamic scale models like Ellington's model (Ellington, 1997) or Dickinson's model (Dickinson, 1999). Dickinson uses a larger wing, which is submerged in a viscous fluid. Flapping frequency is reduced significantly, while maintaining the Reynolds number typical for *Drosophila*.

Table 1 gives an overview of flapping mechanisms which are found in literature and which are either used for experimental research or implemented in FNAVs. These flapping mechanisms all use a single wing (or single pair of wings) and they are designed to mimic a biological flapping wing motion.

**Table 1.**
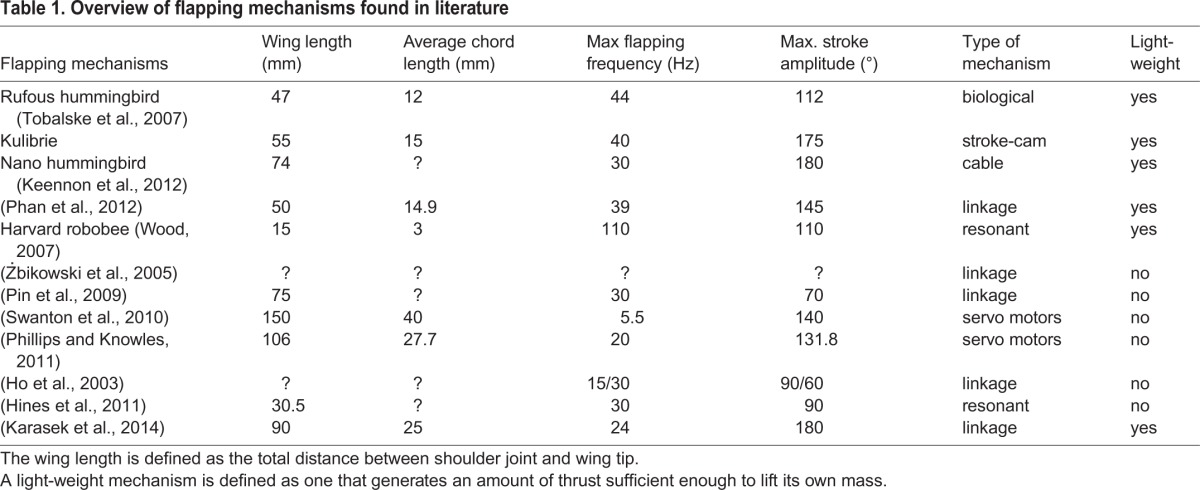
**Overview of flapping mechanisms found in literature**

## RESULTS

### Wing kinematics

**Fig. 2.**
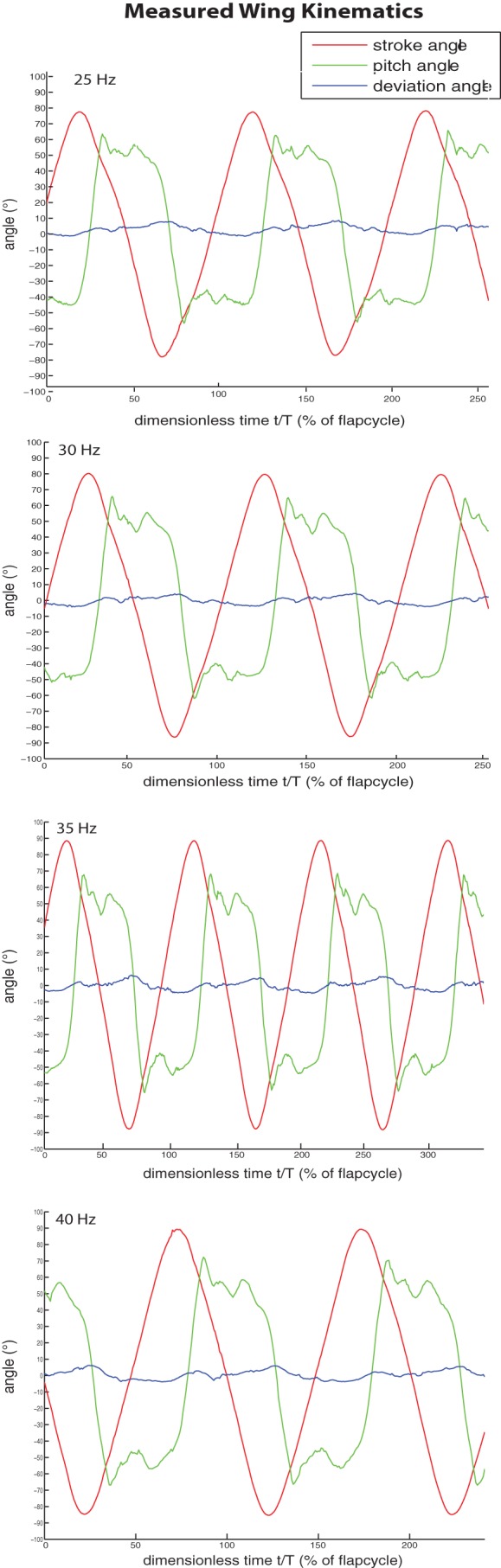
**The measured wing kinematics.** The time course of the stroke angle (red), the deviation angle (blue) and the pitch angle (green) for all experiments.

Fig. 2 shows the variations with time of the stroke angle, the deviation angle and the pitch angle for all experiments. The comparison of Fig. 2 with Fig. 8 shows a remarkable similarity between the wing motion generated with the stroke-cam mechanism and the proposed simplified wing kinematics.

An important observation is that the measured stroke angle, deviation angle and pitch angle are well-repeated over several successive cycles, which means that the mechanism operates accurately. The main parameters for the wing motion are summarised in Table 2.

**Table 2.**
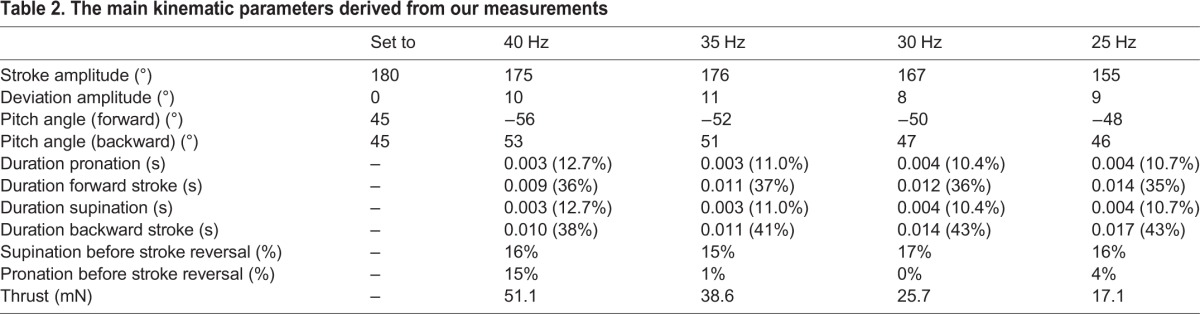
**The main kinematic parameters derived from our measurements**

#### Stroke angle

The measured stroke angles closely approximate the desired harmonical pattern. However a small undesired asymmetry between forward and backward stroke can be distinguished. This asymmetry may be the result of the asymmetry of the friction between the stroke cam and cables described above. The stroke amplitude tends to increase with increasing flapping frequency. We assume this is mainly caused by an elastic deformation of the cable.

#### Deviation angle

Although no deviation of the wing from the stroke plane was intended, the measurements do show a deviation, but with a relatively small amplitude. This deviation is due to the flexibility of the leading edge of the wing and manufacturing tolerances of the rotational joints.

#### Pitch angle

The variation with time of the pitch angle correlates well with the simplified model proposed above. During stroke reversal, which at all flapping frequencies lasts about 10-15% of a flapping cycle, the pitch angle reverses rapidly and almost linearly in time while in between two stroke reversals the measured pitch angle does not change much. The pitch angle reaches a peak value just at the end of each stroke after which it undergoes two or three oscillations before the next stroke reversal takes place.

A small and unintentional difference in pitch angle during forward stroke and pitch angle during backward stroke is measured. This asymmetry may be the result of small asymmetries in the hand-made prototype. On the other hand, pattern repetition is quite precise as each cycle has a variation that is almost identical to other cycles.

### Influence of the flapping frequency on the wing kinematics

**Fig. 3.**
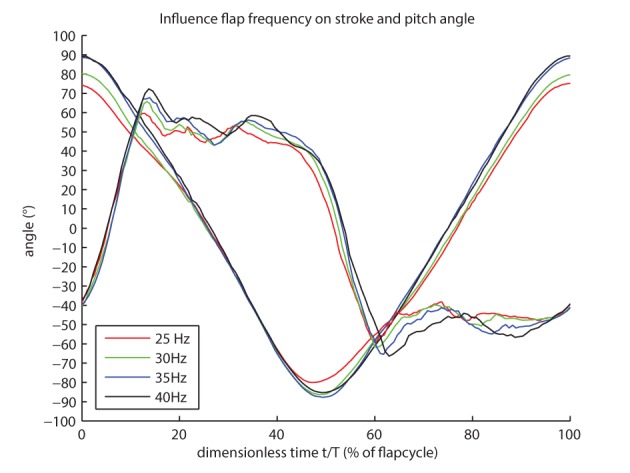
**The influence of the flapping frequency on the stroke and pitch angles.** The time course of the stroke angle and pitching angle for flapping frequencies from 25* *Hz to 40* *Hz shows the influence of the flapping frequency on the stroke amplitude and the angle of attack during midstroke.

Fig. 3 brings together the measured time profiles of the stroke and the pitch angles for each of the four flapping frequencies. The profiles are highly similar, yet with some variations in amplitude and in the instant of time when a peak value is reached. Ideally it should be possible to change the flapping frequency independently of the other kinematic parameters. However Fig. 3 shows that the flapping frequency does have a considerable influence on the stroke amplitude and the pitch angle during forward and backward stroke. Because the exact stroke amplitude and the pitch angle during forward and backward stroke seem to depend on the flapping frequency some tuning is required before they can be set to a desired value in advance.

#### Influence of the flapping frequency on the stroke amplitude

Table 2 shows the kinematic parameters derived from all experiments. From 25* *Hz to 35* *Hz the stroke amplitude increases with increasing flapping frequency, while at 40* *Hz the stroke amplitude is approximately equal to the stroke amplitude at 35 Hz.

#### Influence of the flapping frequency on the pitch angle during forward and backward stroke

The pitch angle during both forward and backward stroke decrease with increasing flapping frequency. The high speed images used to record the wing motion show that this is mainly caused by the elastic deformation of the wing.

### Thrust

Table 2 shows the cycle averaged thrust generated. It ranges from 17.1 mN to 51.1 mN depending on the flapping frequency that is imposed.

As mentioned above, the exact stroke amplitude and pitch kinematics are also affected by the imposed flapping frequency. To quantify the influence of the main kinematic parameters on the thrust independently, more measurements are needed.

## DISCUSSION

### Comparison of the stroke-cam mechanism with previous work in the literature

Table 1 shows a comparison of some characteristics of the wing kinematics generated by the stroke-cam mechanism with the kinematics which are generated by other flapping mechanisms found in literature. A precise comparison of flapping mechanisms is difficult because of the difference in size between the wings which are mounted on each of them. The stroke-cam mechanism is the only light-weight flapping mechanism that is able to generate a wing motion with both a high flapping frequency and a large stroke amplitude. Furthermore, it is capable of generating a harmonic stroke motion. It is also one of the few flapping mechanisms which uses cables as part of the driveline, and which has the advantage of limited inertias and friction in joints.

### Comparison of the resulting wing motion generated by the stroke-cam mechanism with the wing motion of the Rufous hummingbird

**Fig. 4.**
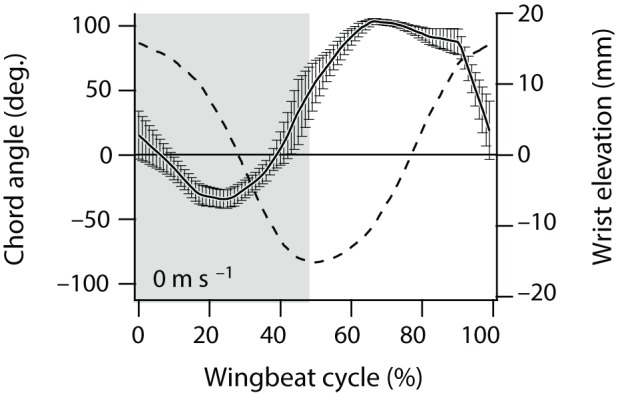
**The measured wing motion of a Rufous hummingbird.** The time course of the wrist elevation (relative to the stroke angle) and the chord angle (relative to the pitch angle) expressed as a percentage of the wing beat cycle (reproduced with permission from Tobalske et al., 2007).

Fig. 4 shows the wing kinematics of a Rufous hummingbird (Tobalske et al., 2007). The wrist elevation in Tobalske's measurements is proportional to the stroke angle of the stroke-cam mechanism such that a wrist elevation of 16* *mm corresponds to a stroke angle of 56°.

The chord angle of Tobalske's measurements is related to the pitch angle of the stroke-cam mechanism. Taking a body angle of 51° and a tracking stroke plane angle of 14° (Tobalske et al., 2007) we find that the chord angle is 35° minus the pitch angle, which gives a pitch angle of −67° during upstroke (chord angle of 92°) and 63° during down stroke (chord angle of −38°). This relationship is only valid during hovering flight. During forward flight the Rufous hummingbird changes its wing kinematics considerably, in particular the stroke plane angle (Tobalske et al., 2007).

#### Flapping frequency

The flapping frequency generated by the stroke-cam mechanism (40* *Hz) nearly equals the flapping frequency of the hovering Rufous hummingbird (44* *Hz) (Tobalske et al., 2007), but it is considerably smaller than the flapping frequency (56* *Hz) measured on a Rufous hummingbird lifting maximal load (Altshuler et al., 2010).

#### Wing morphology

The wing we developed and used in our experiments does not show a significant difference in camber between forward and backward stroke in contrast to a real hummingbird wing which does show a significant difference in camber between forward and backward stroke. As a consequence the wing motion necessary to hover is symmetrical in forward and backward stroke, in contrast to the motion of a real hummingbird.

#### Stroke plane

Due to the wing symmetry and the symmetry in wing kinematics of forward and backward stroke, the stroke plane of the stroke-cam flapping mechanism has to be oriented horizontally to hover. The Rufous hummingbird slightly tilts it stroke plane (14°) during hovering (Tobalske et al., 2007).

#### Stroke amplitude

The stroke-cam flapping mechanism generates a stroke amplitude of 175°, considerably larger than the stroke amplitude of 112° measured on a hovering Rufous hummingbird (Tobalske et al., 2007), but smaller than the stroke amplitude measured on a Rufous hummingbird lifting maximal load (Altshuler et al., 2010).

#### Pitch angle during forward stroke and backward stroke

A small and unintentional asymmetry exists in pitch angle during forward stroke and backward stroke for the wing motion generated by the stroke-cam mechanism at 40* *Hz (−56° vs 53°) (Table 2). This asymmetry is smaller than the asymmetry measured on a Rufous hummingbird (−67° vs 63°) (Tobalske et al., 2007).

#### Maximal load lifting capability

The load lifting capability of the stroke cam flapping mechanism (5.1* g*) is larger than the one generated by a Rufous hummingbird [3.97* g* for each wing (Altshuler et al., 2010)]. This difference is remarkable, because both the flapping frequency and the stroke amplitude measured on the Rufous hummingbird under maximal load lifting are considerably larger. This difference in load lifting capability may be caused by a small difference in wing length but it is probably also a result of the differences in wing morphology and kinematics as described in the sections Wing morphology, Stroke plane, Stroke amplitude and Pitch angle during forward stroke and backward stroke. It is known from particle image velocimetry measurements that a Rufous hummingbird generates considerably more thrust during forward stroke than during backward stroke (Warrick et al., 2005).

We believe that the inability of the Rufous hummingbird to generate an optimized symmetrical wing stroke motion in forward and backward stroke is due to biomechanical constraints, like the difference in wing camber between forward and backward stroke, to its load lifting capability.

#### Motors mass versus muscle mass

This section compares the characteristics of an artificial bird that uses the stroke-cam mechanism to different species of birds in nature. A hummingbird-like robot would use a set of two stroke-cam mechanisms and two motors as presented in this paper. The load-lifting capability of this robot would be 10.22* g* (5.11* g* per wing). The mass of the two motors combined would be 3.28* g*, or 32% of the maximum load lifting capability. This robot would have a wingspan of about 125* *mm (two times a 55* *mm wingtip-to-shoulder distance and a distance between the two shoulders of about 15* *mm).

The *Ocreatus underwoodii* (slightly smaller wing span) has a comparable load-lifting capability of 9.99* g*. This species has a flight muscle mass (defined as the summed mass of the pectoralis major and the supracoracoideus) of 0.633* g*, which is 21% of its body mass (3.052* g*) (Altshuler et al., 2010). It is clear from this comparison that the motor mass of a hummingbird-like robot which uses two stroke-cam mechanisms is considerably larger (about five times) than the muscle mass of a hummingbird with a comparable load lifting capability.

In addition it should be mentioned that at this point no means to perform flight manoeuvres are yet implemented. Implementing this ability would probably increase the total motor mass.

### Conclusions

This paper presents the concept of a drive system for a hummingbird-like robot with flapping wings in hovering flight, with the Rufous hummingbird having served as a reference. The core of the development is the stroke-cam mechanism which generates a reciprocating motion of the wing. The paper further compares the artificial wing kinematics to the real bird.

The stroke amplitude and angle of attack at midstroke are found to depend on the flapping frequency.

At this moment we only studied hovering kinematics. In the future it would be interesting to study forward flight and to study how flight manoeuvres could be performed and compare these results with the forward flight kinematics and flight agility of hummingbirds.

## MATERIALS AND METHODS

### Simplified wing morphology and kinematics

By only taking into account the features of the wing morphology and motion which have a considerable influence on the thrust as described above, the wing motion can be simplified by leaving some particular features which do not contribute much to the level of thrust which is generated. As a consequence, mechanical design of the flapping mechanism is somewhat simplified.

#### Wing morphology

**Fig. 5.**
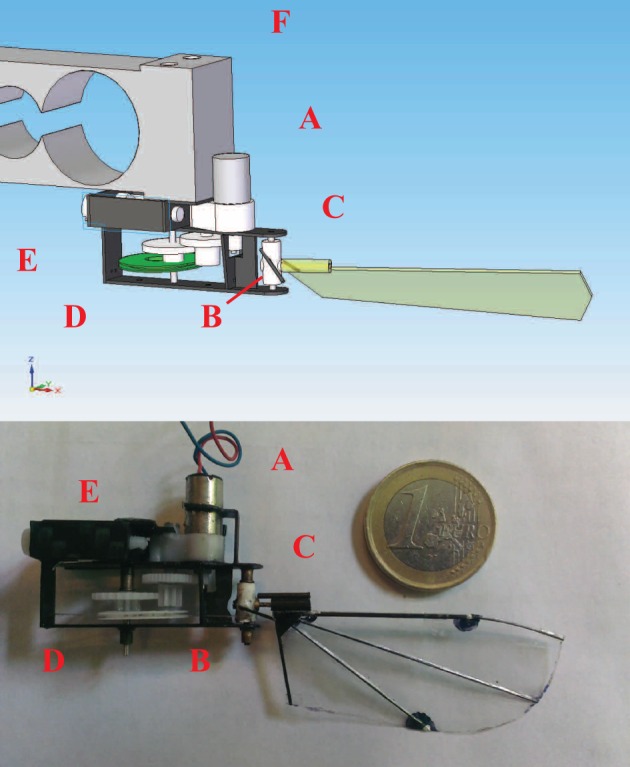
**The practical implementation of the stroke-cam mechanism.** DC motor (A), pitch blocking elements (B) black rods mounted on the wing wheel (C), stroke-cam (D), screws to adjust tension in the cable (E), and 1668S load cell from BCM (F).

Fig. 5 shows the wing shape which is used in our experiments. The shape of the wing is based on the work of Ellington (1984). The wing length is 50* *mm and the average chord length is 18* *mm. The leading edge and veins are pultruded carbon composite rods, all attached to each other near the shoulder joint. The shoulder joint allows the wing to pitch around its leading edge. The wing membrane is a polyester film with a thickness of 0.015* *mm. This design results in a stiff wing that, unlike a real hummingbird wing, has no difference in camber between forward and backward stroke.

#### Simplified wing kinematics

Three angular degrees of freedom are sufficient to fully describe the motion of a stiff robotic hummingbird wing relative to its body as shown in Fig. 1. The wing tip follows a figure-of-eight trajectory. The z-axis is taken to be perpendicular to the average stroke plane (grey), which goes through the centre of this figure-of-eight trajectory; the x-axis is taken to be perpendicular to the z-axis and is parallel to the wings at midstroke, when both wings are in line with each other. The y-axis is taken to be perpendicular to the x- and z-axes.

**Fig. 6.**
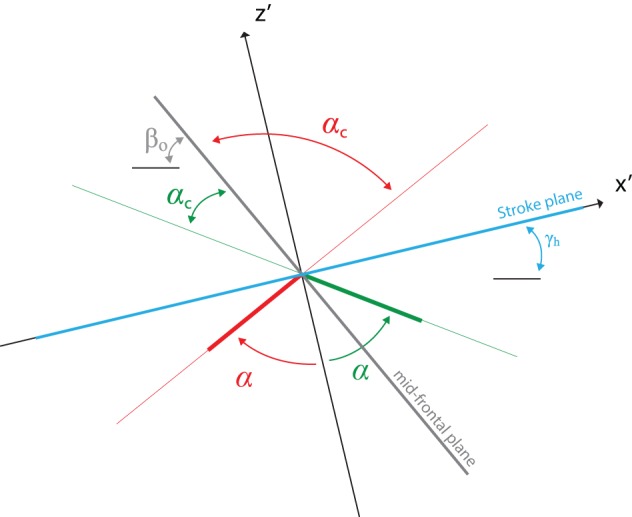
**The pitch angle and it relation to the chord angle.** This figure depicts a plane perpendicular to the leading edge of the wing. The x′ axis is taken horizontally and the z′ axis perpendicular to the x′ axis. The pitch angle (α) is negative during upward stroke (red) and positive during backward stroke (green). The pitch angle can be derived from the chord angle, knowing the tracking stroke plane angle (γ_h_) and the body angle (β_o_).

The stroke angle parameter *ϕ* measures the orientation of the wing in its back and forth stroking (sweeping) motion, it is defined as the angle between the x-axis and the projection of the wing's leading edge onto the stroke plane. The deviation angle parameter *β* measures the deviation (plunging) of the wing out of the stroke plane and it is defined as the angle between the wing's leading edge and its projection onto the stroke plane. The pitch angle parameter *α* measures the wing rotation about its leading edge. The pitch angle is defined in a plane perpendicular to the leading edge as shown in Fig. 6, it is zero if the wing is perpendicular to the stroke plane. In this figure the x′ axis is taken to be horizontal and the z′ axis perpendicular to the x′ axis.

The stroke range is defined as the angle between the minimum and maximum stroke angles and the deviation range as the angle between the minimum and maximum deviation angles.

**Fig. 7.**
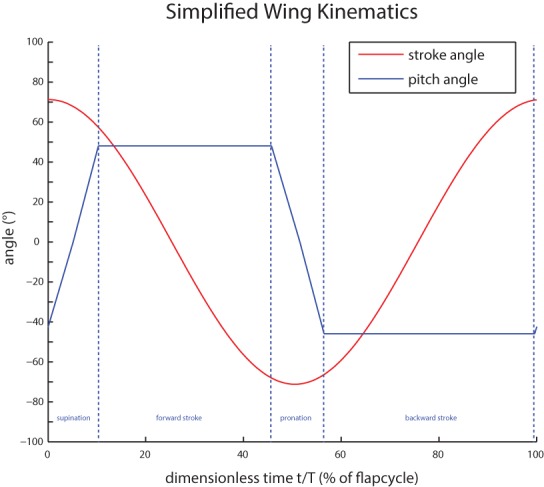
**The simplified model for the wing motion.** The deviation is neglected. The stroke angle is approximated to vary harmonically in time. The pitch angle is approximated by a piecewise linear function representing the four phases of the wing motion. During supination and pronation the pitch angle is approximated to vary linearly in time while during the forward and backward stroke the pitch angle is assumed to be constant.

To simplify the design of the flapping mechanism we take the following assumptions to obtain simplified wing kinematics:

1. The deviation can be neglected and does not need to be actively controlled.
2. The stroke plane can be taken horizontally.
3. The stroke angle varies approximately harmonically in time.
4. The variation of the pitch angle in time can be approximated as in Fig. 7 by a piecewise linear function according to the four phases of the wing motion. During supination and pronation the pitch angle is assumed to vary linearly with time while during the forward and backward stroke the pitch angle is assumed to be constant. It is called respectively the pitch angle during forward and the pitch angle during backward stroke.

To quantify the extent to which an advanced wing rotation occurs, we define the supination/pronation before wing reversal as the percentage of the total duration of the supination/pronation that occurs before stroke reversal. A supination/pronation before wing reversal above 50% depicts an advanced wing rotation.

### The stroke-cam flapping mechanism

The design of a lightweight flapping mechanism which reproduces the simplified wing motion of a small hummingbird as described above is challenging because of some specific requirements:

1. The stroke amplitude needs to be sufficiently large; at least 112° like that of a Rufous hummingbird (Tobalske et al., 2007). A larger stroke amplitude is preferred because it results in a larger wing speed and thus an increased thrust. The maximum stroke amplitude is limited to somewhat less than 180° to prevent the wings from colliding to each other while maintaining some margin of safety.
2. The stroke angle needs to vary harmonically to minimise peaks in acceleration and jerk of the wing load.
3. The flapping frequency needs to be sufficient high, like that of a Rufous hummingbird (44* *Hz) (Tobalske et al., 2007), but preferably higher. This requirement translates into low inertia of all accelerating parts and minimum friction losses in all articulations.
4. The mass of the total system including the frame, flapping mechanism, motor, energy source and driving electronics must not exceed the thrust which is generated by the flapping wing in order to be used in an FNAV.
5. The mechanism should be robust enough to be able to work properly during several minutes of flight.

Many different lightweight flapping mechanisms are described in literature (Table 1). They typically use linkage mechanisms to convert the motion of the rotating shaft of a small electric motor to a reciprocating motion of the wing. Traditional linkage mechanisms have two major disadvantages; they are unable to generate a sufficiently large stroke amplitude nor can they generate a pure harmonic stroke motion.

The next section describes a new stroke-cam flapping mechanism. The stroke-cam mechanism generates a harmonic stroke motion with a stroke amplitude which may be as large as desired (not exceeding the 180° limit with some margin).

#### Stroke

**Fig. 8.**
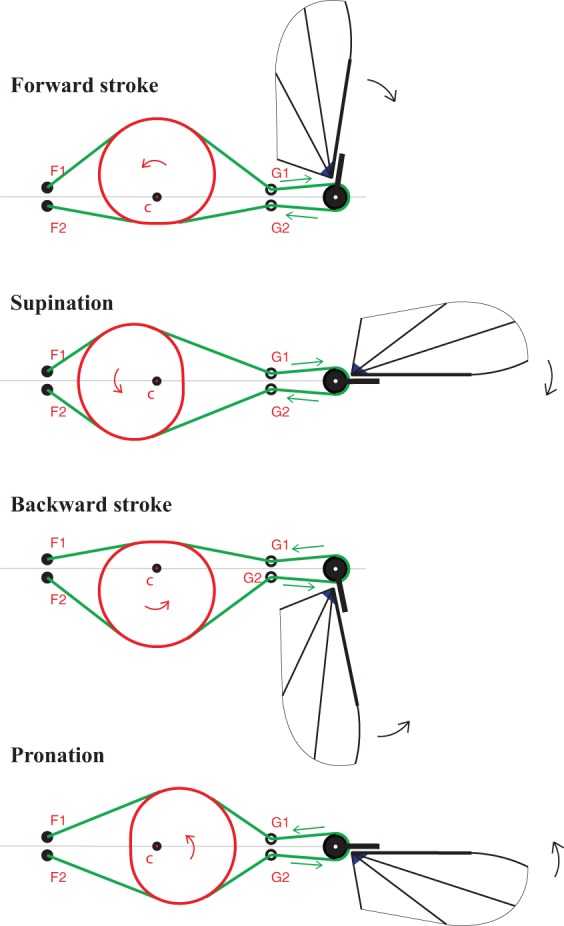
**The stroke-cam mechanism**. The stroke-cam (red) rotates around C and it pushes the cables (green) in such a way that they pull at the wing wheel (black) in an alternating way, resulting in the stroke motion of the wing.

The central element in the mechanism is a cam, which is designed with a particular cam profile. A static cable, which is mounted in the frame, slides over the cam and the rotating cam drives a pulley via the cable. The stroke-cam mechanism (illustrated in Fig. 8) converts the motion of a rotating axis of a small electric motor to reciprocating motion of a wing. The stroke-cam mechanism consists of a stroke-cam (red) which rotates around *C*, two cables (green) which are fixed at both extreme ends and which slip over the stroke cam and a wing wheel (black) which rotates around its centre. At one side of the stroke cam the cables are fixed at points *F1* and *F2* and on the other side of the stroke cam the cables are guided through guides *G1* and *G2* and fixed to the wing wheel. The wings are attached to this wing wheel by means of a joint which allows for the pitching of the wing. The cables are pre-tensioned.

When the stroke cam rotates around *C* it moves the cables in such a way that they pull alternatingly at the wing wheel resulting in the stroke motion of the wing. The cables are pre-tensioned such that compressive forces can be transmitted during the whole flapping cycle.

Both the shape of the stroke-cam and the relative coordinates of *F1*, *F2*, *G1* and *G2* with respect to *C* have an influence on the course of stroke. To generate a stroke that closely approximates the desired harmonic course the shape of the stroke cam can be defined in polar coordinates as:
(1)
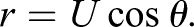
In Eqn 1, with *C* as the origin, *r* is the distance to *C* and *θ* is the angle from the horizontal direction to the right (Fig. 8). *U* is a scaling factor. The coordinates of points *F1*, *F2*, *G1* and *G2* are then be defined relative to *C* and in function of *U* as follows:
(2)
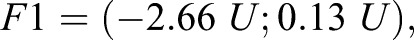

(3)
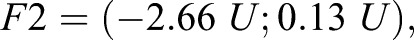

(4)
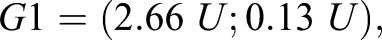

(5)
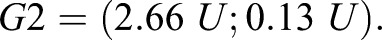


The stroke-cam mechanism generates a stroke motion that closely approximates a harmonic evolution of the stroke angle *ϕ* with time. Theoretically the course of the stroke is symmetric. However, as can be seen in Fig. 8, during backward stroke and supination the cables move in the same direction as the stroke cam whereas during forward stroke and pronation the cables move in the opposite direction of the stroke cam resulting in a variation in friction between cables and stroke cam which may theoretically result in a slightly asymmetric driving effect on the wing wheel. Measurements on the mechanism indeed reveal some degree of asymmetry.

The stroke amplitude generated with the stroke-cam mechanism is proportional to the radius of the wing wheel. This mechanism drives one degree of freedom, the stroke angle *ϕ*.

#### Pitch

The second degree of freedom is the pitch angle, which is not actively controlled. The wing is attached to the wing wheel by means of a rotational joint. This enables the wing to pitch passively around its leading edge under the influence of the aerodynamic forces acting on the wing and the inertia of the wing. To obtain a desired pitch angle during forward and backward stroke we constrain the pitching motion with two pitch blocking elements (black), shown in Fig. 5.

#### Adjustability of the Kinematic Parameters

The kinematic parameters that can be modified to alter the flapping motion are the flapping frequency, stroke amplitude and the pitch angle during forward and backward stroke.

The flapping frequency can be continuously varied during operation by changing the input voltage to the motor. Changing the flapping amplitude requires replacing the stroke cam with one of a different size. Changing the maximum pitch angle during forward and backward stroke requires repositioning the pitching blocking elements.

### Practical implementation

Fig. 5 shows the practical implementation of the stroke-cam mechanism. The flapping mechanism is driven by a coreless brushed DC motor with a diameter of 6* *mm through a 3 gear transmission with a total gear ratio of 14.75. The stroke cam material is ABS and it is printed with a SST 1200ES printer. The cables are nylon cables with a diameter of 0.1* *mm. The tension in the cable can be adjusted with two small screws. The frame is assembled from several elements cut out of a 0.5* *mm carbon composite plate which are consecutively glued together. The total mass of this setup is 3.39* g* of which the wing constitutes 0.048* g* and the motor constitutes 1.64* g*.

### Experimental validation of wing kinematics and thrust generation

To validate the resulting wing motion and the thrust generated by the stroke-cam flapping mechanism a set of experiments are performed in which the stroke amplitude is set to 180° and the pitch angle during forward and backward stroke to 45°. Experiments are conducted at several values for the flapping frequency which is successively set at 25* *Hz, 30* *Hz, 35* *Hz and 40 Hz to examine its influence on the other kinematic parameters. The flapping mechanism is mounted on to the load cell (Fig. 5) so that the component of force which is perpendicular to the stroke plane is measured. This force component is defined as the thrust generated by the flapping wing.

#### Measuring the wing kinematics

**Fig. 9.**
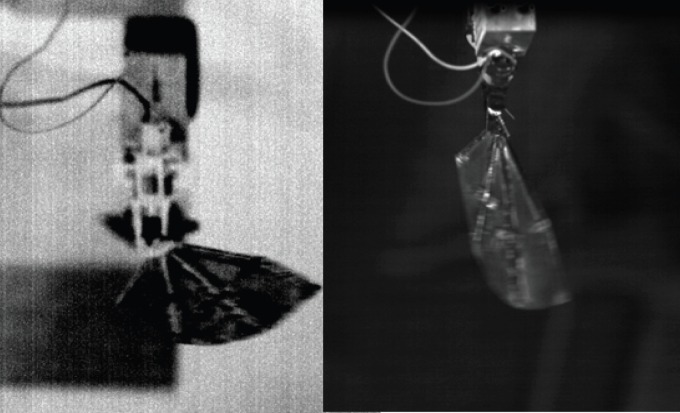
**High speed images of the flapping wing.** Left: front view of the flapping wing. Right: top view of the flapping wing. These images are taken at a frame rate of 5000* *Hz.

The wing motion is measured by tracking three markers on the wing, two markers on the leading edge and one on the trailing edge as shown in Fig. 5. The markers are tracked using two HighSpeedStar 6 cameras from LaVision (http://www.lavision.de/en/products/cameras/high_speed_cameras.php) in a stereo set-up. One camera takes top view images and the other camera takes front view images (Fig. 9). Three spotlights are used to illuminate the moving wing.

The recordings are done at a frame rate of 5000* *Hz and post-processed using DigitizingTools, a free software package provided by the Hedrick Lab (http://www.unc.edu/~thedrick/software1.html). After tracking the wing motion the tracking records are further analysed in Matlab to generate stroke, pitch and deviation angles as shown in Fig. 2.

#### Thrust measurement

The thrust is measured 200 times per second by a common double beam strain-gauge load cell (model 1668S from BCM) and averaged over a period of 5 s. The load cell is connected to a Scaime CPJ measurement bridge with built-in amplifier. The measurement accuracy is 0.5 mN.
